# Glucocorticoid Receptor Localizes to Adherens Junctions at the Plasma Membrane of Keratinocytes

**DOI:** 10.1371/journal.pone.0063453

**Published:** 2013-04-30

**Authors:** Olivera Stojadinovic, Andrew Sawaya, Irena Pastar, Marjana Tomic-Canic

**Affiliations:** 1 Wound Healing and Regenerative Medicine Research Program, Department of Dermatology and Cutaneous Surgery, University of Miami Miller School of Medicine, Miami, Florida, United States of America; 2 Cellular and Molecular Pharmacology Graduate Program in Biomedical Sciences, University of Miami Miller School of Medicine, Miami, Florida, United States of America; Cornell University, United States of America

## Abstract

Glucocorticoids are important regulators of epidermal tissue homeostasis. As such, their clinical applications are widespread, ranging from inflammatory skin disorders to keloids and cancer. Glucocorticoids exert their effect by binding to glucocorticoid receptor (GR) which translocates to the nucleus and regulates gene expression (genomic effect). In addition, GR has rapid non- genomic effects that are mediated by cell signaling proteins and do not involve gene transcription. Although genomic effects of GR in the epidermis are well documented, the non-genomic effects are not completely understood. Therefore, we utilized immunostaining and immunoprecipitations to determine specific localization of the GR in human keratinocytes that may contribute to non-genomic effects of glucocorticoid action. Here we describe a novel finding of GR localization to the plasma membrane of keratinocytes. Immunocytochemistry showed co-localization of GR with α-catenin. Immunoprecipitation of the membranous fraction revealed an association of GR with α-catenin, confirming its localization to adherens junctions. We conclude that GR localization to adherens junctions of keratinocytes provides a new mechanism of non-genomic signaling by glucocorticoids which may have significant biological and clinical impact.

## Introduction

Glucocorticoids play an important role in a variety of dermatologic conditions ranging from wound healing to psoriasis as well as being a mainstay of therapy in clinical Dermatology. Traditionally, their effects are mediated through glucocorticoid receptor (GR) which is known as a ligand-activated transcription factor [1]. GR is present in the cytoplasm in its inactive form, bound to the heat shock protein Hsp90 [2]. Hormone binding causes activation of the receptor, release from Hsp90, and its translocation to the nucleus. Activated GR complexes with multiple proteins (co-regulators) binds to promoter sequences and regulates transcription. Our laboratory and many others have studied mechanisms by which GR regulates transcription of genes important for skin physiology and pathology [1]–[5].

More recently, the non-genomic effects of steroid receptors, including GR, estrogen, and progesterone receptor, have been elucidated [6]–[8]. These very rapid effects are mediated within minutes and do not involve direct GR binding to the promoter sequences [9], [10]. GR regulates multiple signaling pathways including MAPK, cAMP-PKA, PI3K, and PLC-PKC pathways in a variety of tissue types such as neuronal and erythroid cells [8], [11], [12]. A subset of the non-genomic effects of GR is thought to be mediated by an interaction between G protein-coupled receptors (GPCRs) and membranous GR [7], [8], [13], [14].

The localization of GR in the cell and the exact mechanism by which it exerts its non-genomic effects in skin remains unknown. One possible mechanism by which GR could interact with membrane receptors or signaling proteins to exert its rapid effects would be to localize to the plasma membrane. Therefore, we investigated if GR localizes to the plasma membrane and which membrane-bound proteins are associated with it. We found that the GR localizes to the plasma membrane of primary human keratinocytes. We observed that it co-localizes with α-catenin suggesting its association with adherens junctions (AJ). To the best of our knowledge this is the first observation of GR membranous localization and its association with AJ component, alpha-catenin.

## Results

**Figure 1:**
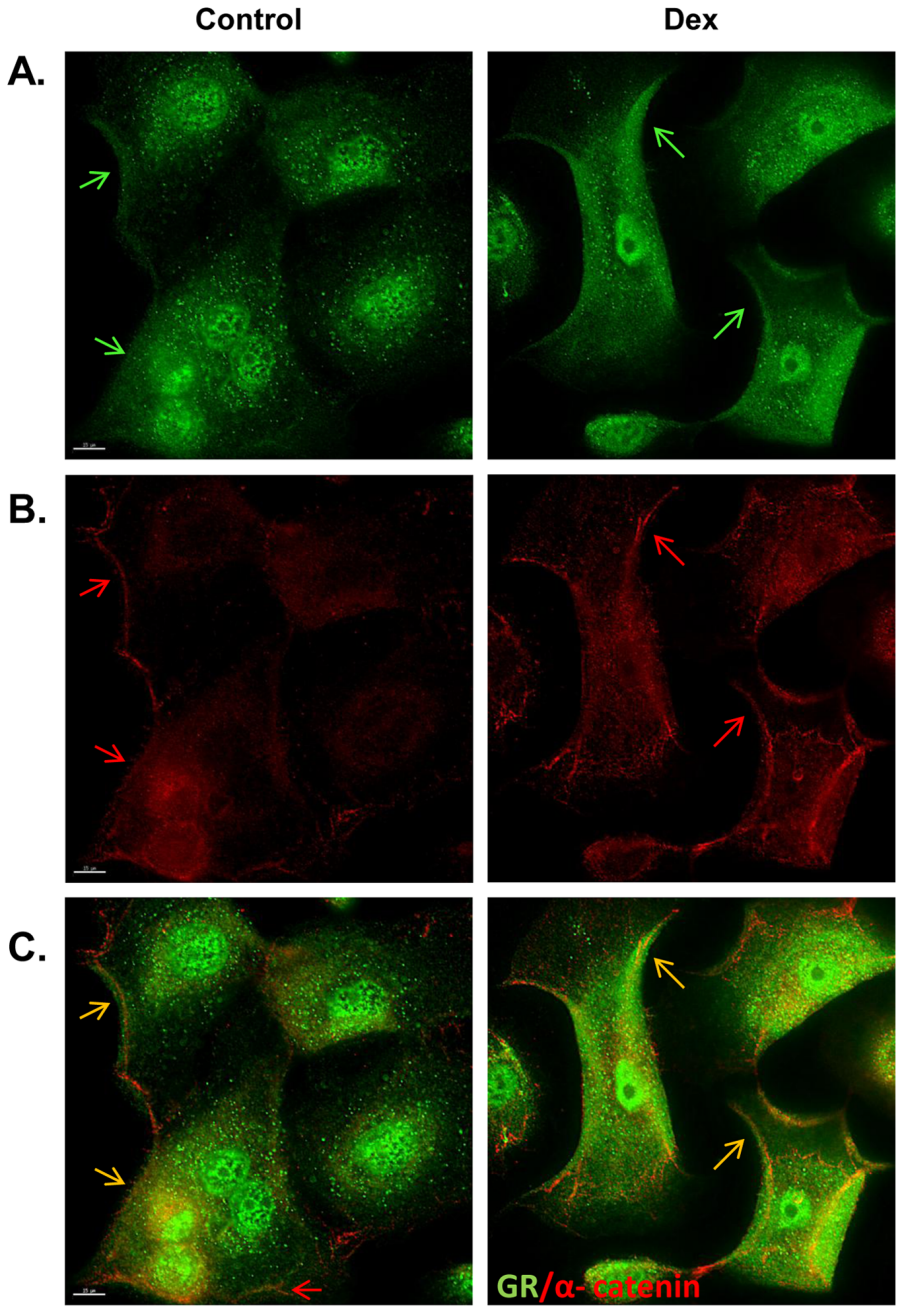
Co-localization of the GR and α-catenin to the plasma membrane of keratinocytes. Immunofluorescent staining of primary human keratinocytes incubated in the absence or presence of DEX shows co-localization of GR and α-catenin to the plasma membrane. Green fluorescence indicates presence of GR (A); red alpha-catenin (B) and yellow merge (C). Arrows point to the membranous staining. Scale bar 15 µm.

As initial step to determine the localization of the glucocorticoid receptor (GR) in primary human keratinocytes we used immunohistochemistry. Primary human keratinocytes were treated with 1 μM of dexamethasone (Dex) for 30 minutes and upon fixation stained with GR-specific antibody to determine the cellular localization of the receptor. In addition, we used α-catenin specific antibody to mark the plasma membrane. As expected, GR was found in the cytoplasm and nucleus of the keratinocytes (**Figure 1**). Interestingly, we also found a fraction of GR that was localized on the plasma membrane (**Figure 1A** **; C**). Upon further examinations we observed that GR co-localized, in part, with α-catenin (**Figure 1B** **; C**). This suggests that GR may be associated with adherens junctions (AJ).

**Figure 2:**
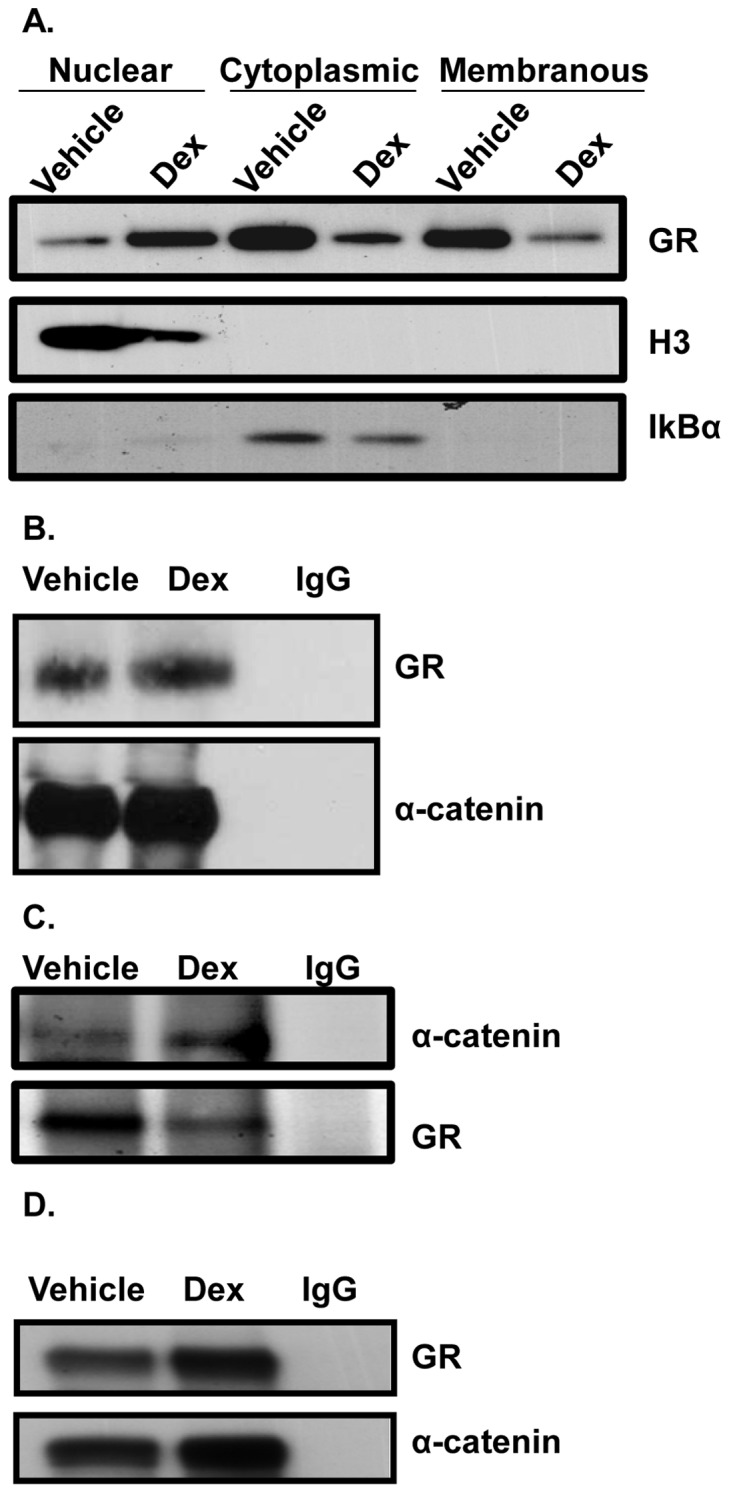
GR is bound to α-catenin in the plasma membrane of keratinocytes. (**A**) Cell fractionation was performed on primary human keratinocytes following 30 min treatment with Dex or vehicle (V). The purity of the fractions was confirmed using: histone H3 or IkBα specific antibodies. H3 was found only in nuclear fractions whereas IkB α was found only in cytoplasmic fractions. (**B**) Immunoprecipitations following a treatment of keratinocytes with Dex or vehicle were performed with an antibody against α-catenin. The immunoprecipitation was then immunoblotted with GR. (**C**) Conversely, immunoprecipitation was performed with an antibody against GR. The immunoprecipitation was then immunoblotted with α-catenin. (**D**) Immunoprecipitations following a treatment of human epidermis with vehicle or Dex was performed with an antibody against α–catenin, and immunoblotted with GR. Normal IgG was used as a control.

Next, we performed cellular fractionations and Western blotting to establish the presence of GR in various cellular compartments. To test how presence of hormone influences GR localization primary human keratinocytes were incubated in the presence or absence of Dex. The purity of the fractions were confirmed as follows: histone H3 was found only in nuclear fractions whereas it was absent from cytoplasmic and membranous (**Figure 2A**). Conversely, IkBa was found only in cytoplasmic fractions whereas it was absent from either nuclear or membranous. Interestingly we found GR present in all three cellular compartments (**Figure 2A**). As expected, nuclear presence of GR markedly increased upon hormone treatment, whereas cytoplasmic significantly decreased, confirming hormone-dependent translocation from cytoplasm to the nucleus. Furthermore, GR maintained its presence in membranous fraction in both conditions. Membranous GR was decreased in the presence of hormone, suggesting that membranous GR may also contribute to nuclear GR. Importantly, continuous presence of GR in the membranous fraction supports the notion of the association of GR to the plasma membrane.

GR is known to inhibit wound healing by disrupting migration of keratinocytes [1], [5]. The co-localization of GR and α-catenin raised an intriguing possibility that membranous GR may be associated with adherens junctions (AJ), especially because cell-cell interactions play an important role in maintaining both migration and proper skin barrier formation. To test GR interaction with AJ components, we performed co-immunoprecipitation studies. Interestingly, GR was found in a complex bound to α-catenin (**Figure 2B**). Next, we performed the converse experiment. This time we used GR-specific antibody to pull down GR-bound proteins from the membranous fraction. As expected, GR was found present in both fractions, although it showed slight decrease upon incubation with hormone (**Figure 2C**). Next we probed the same membrane with α-catenin specific antibody and found presence of α-catenin in both fractions. GR-associated α-catenin shows slight increase in the fraction obtained from cells incubated with hormone. Further, we examined whether GR interacts with α-catenin in human epidermis. Upon incubation of human skin in the presence or absence of Dex, the epidermis was separated from the dermis, proteins were isolated, followed by immunoprecipitation with α-catenin specific antibody to pull down α-catenin bound proteins. GR was found in a complex bound to α-catenin recapitulating the findings observed in primary human keratinocytes (**Figure 2D**). We conclude that the interaction between GR and α-catenin occurred in the presence and absence of the hormone, suggesting GR association to adherens junctions in mediating constitutive, non-genomic effects.

Our initial observation leads to conclusion that GR localizes to the plasma membrane of keratinocytes as an element of adherens junctions, in association to α – catenin.

## Discussion

To the best of our knowledge, this is the first evidence of GR localization to the plasma membrane of human epidermal keratinocytes. This has important implications for understanding the rapid effects of GR. Elucidating the membrane receptors and signaling proteins that associate with GR in the membrane provides the opportunity to determine novel signaling pathways activated by GR in skin. Other steroid receptor variants localize to the plasma membrane as well. A splice variant of estrogen receptor specifically localizes to the membrane and plays an important role in signaling and cancer [15], [16]. We found full size GR in the membranous fraction.

Our data are in agreement with previous observations of GR cytoplasmic and nuclear localization [2], [17], [18]. It is well established that upon hormone treatment, the majority of GR translocates from the cytoplasm to the nucleus, which is evident from our cell fractionation experiments. However, there is a significant amount of GR present in the nuclear and membranous fractions in addition to cytoplasmic, in the absence of the hormone, strongly supporting recent findings of our laboratory and others of constitutive endogenous synthesis of cortisol in keratinocytes [19]–[22]. Thus, in the absence of exogenous hormone a fraction of receptor is steadily present in the keratinocyte nuclei and associated with plasma membrane, constitutively mediating its genomic and non-genomic effects respectively.

Interestingly, GR localization to the plasma membrane and its association with α-catenin is maintained in the presence of the ligand. Decrease of GR in the membranous fraction is similar to its decrease from the cytoplasmic compartment, suggesting that membranous GR may also contribute to the nuclear portion of the GR. Interestingly, hormone treatment increased association of α-catenin in the membranous fraction that was immunoprecipitated by GR-specific antibody, indicating that perhaps hormone-mediated conformational change of the GR may strengthen the α-catenin-GR complex. Flow through following immunoprecipitation of membranous fraction using GR antibody shows also presence of α-catenin, suggesting that GR is associated with only a fraction of membranous α-catenin (Figure S1), which is not surprising given that GR is signaling rather than a structural protein. A question remains if a constitutive fraction of GR located on the plasma membrane is distinct from classic cytoplasmic/nuclear GR. This could have significant implications for targeted steroid therapies and their side effect profiles. If the membranous GR indeed is distinct from the “classic” GR then it may be possible to target selectively one receptor type and spare the other.

Close association of GR with components of AJs raises interesting possibilities as well. We have shown previously that β-catenin, another component of AJs, acts as co-repressor of GR-mediated transcriptional regulation of keratin genes involved in wound healing [23]. In addition, we have shown recently that cholesterol synthesis participates in glucocorticoid-mediated signaling in the epidermis and that statins may act to reverse GR-mediated inhibition of wound healing [4]. Thus, membranous GR may be involved in multiple signal transduction cascades that have not previously been identified. This is currently under investigation. It is also important to identify what is the role of the membranous and non-genomic effects of GR in dermatologic conditions such as chronic wounds and psoriasis.

In summary, localization of GR to the plasma membrane is a novel finding and has significant implications in understanding the mechanism by which GR exerts its non-genomic effects in skin and the potential for treatment of various GR-associated dermatologic conditions.

## Materials and Methods

### Immunocytochemistry

Primary human keratinocytes [24] were grown to about 40–60% confluence and were incubated for 24 hrs in a basal serum-free custom made medium without hydrocortisone (Invitrogen). The cells were then treated with either vehicle or 1 μM dexamethasone (DEX) as previously described [1]. The cells were then fixed in acetone-methanol (1∶1) for 2 min, permeabilized with 0.1% Triton X-100 for 10 min and incubated overnight at 4°C with GR antibody (Thermo Scientific) and α -catenin antibody (BD Transduction) as a marker of the plasma membrane. Staining was visualized using a secondary fluorescein (AlexaFluor 488) anti-rabbit antibody and anti-mouse (AlexaFlour 594)(Invitrogen) antibodies respectively, as described [1].

Fluorescent images were acquired using the Olympus 1X71 Applied Precision DeltaVision Elite inverted microscope system equipped with a CoolSNAP HQ2 digital camera (Photometrics, Tuscon, AZ) and SoftWorx imaging software version 5.5 (Applied Precision). Images were then deconvolved using a constrained-iterative algorithm in SoftWoRx with an aggressive ratio, ten iteration cycles and medium noise filtering.

### Western Blots and Immunoprecipitation

Extracts for cell fractionation and immunoblotting were prepared from a subconfluent 10-cm plate of primary human keratinocytes treated with vehicle (ethanol) or DEX (1 μM) for 30 min. The cells were placed on ice, washed twice with PBS, and lysed with buffer (10 mM Tris pH 7.4; 2 mM MgCl_2_; 10 mM NaCl; protease/phosphatase inhibitors). Lysates were ran through a syringe and then centrifuged at 2800 g at 4°C for 10 min to isolate the nuclear fraction. The supernatants were obtained and centrifuged at 100,000 g at 4°C for 1 hour to isolate the cytosolic and membranous fractions. The pellets (membranous fraction) were re-suspended in TEB buffer (1% Triton X-100; 250 mM NaCl; 2 mM EGTA; 2.5 mM MgCl_2_; 25 mM Tris pH7.4; protease/phosphatase inhibitors) and immunoprecipitations were carried using 112 μg of protein using the Pierce Classic IP kit (Thermo Scientific) according to manufacturer's instructions using anti-GR (5 μl; Thermo Scientific) or anti- α -catenin (2 μg; BD Transduction), or normal IgG (2 μg; Santa Cruz).

Human skin specimens were obtained as discarded tissue from reduction surgery procedures in accordance to institutional approvals. Specifically, protocol to obtain unidentified, discarded human skin specimens from reduction surgery was submitted to University of Miami Human Subject Research Office (HSRO). Upon review conducted by University of Miami Institutional Review Board (IRB) it was determined that such protocol does not constitute Human Subject Research, since discarded skin specimens do not contain any of the 18 identifiers noted in the privacy rule. As such, this project was not subject to IRB review under 45 CFR46. Since this research activity was determined as non-Human Subject research no informed consent was necessary.

Obtained specimens were treated with either vehicle (ethanol) or Dex (1 μM) in DMEM (Lonza) containg 10% Hyclone FBS (Thermo Scientific) and Penicillin/Streptomycin (Invitrogen) for 2 hours at 37°C. Skin explants were incubated overnight at 4°C in 1.25 U/mL Dispase II (Roche Applied Sciences) to separate the epidermis and dermis. Protein was isolated from epidermis and immunoprecipitated using 500 μg protein with anti-α-catenin (2 μg; BD Transduction), or normal IgG (2 μg; Santa Cruz).

IPs were resolved on 4-20% Criterion TGX pre-cast gels (Bio-Rad), then transferred to polyvinylidene difluoride membranes (Thermo Scientific) and placed in blocking buffer for 1 hour (TBS, 0.05% Tween20, 5% BSA) then probed with anti-GR (1∶1000; Invitrogen) or anti- α -catenin (1∶1000; BD Transduction) diluted in blocking buffer for 1 hour. Membranes were washed three times with TBST and incubated with mouse secondary horseradish peroxidase (HRP)-linked antibodies (Cell Signaling) and developed using Pierce ECL Western Blotting Substrate (Thermo Scientific) according to manufacturer's instructions. Western blots from cell fractions were carried out using 10 μg of protein from each fraction and probed using anti-GR (1∶1000; Invitrogen), anti-Histone3 (1∶2000; Abcam), and anti-IkBα (1∶1000; Santa Cruz) and western blots were carried out as described above.

## Supporting Information

Figure S1 **Immunoprecipitation controls.** Flow through from immunoprecitiptations shown in Figure 2 were blotted for the presence of GR and α-catenin As expected, no remaining GR was found in samples originating from cells incubated with Dex, whereas very little was found in vehicle treated samples. Alpha-catenin was found in both fractions.
(TIF)
