# Supplementary figures and images for: Glucocorticoid Receptor Localizes to Adherens Junctions at the Plasma Membrane of Keratinocytes
Source: PLoS One. 2013 Apr 30;8(4):e63453. doi: 10.1371/journal.pone.0063453 (PMC3639973; doi:10.1371/journal.pone.0063453)

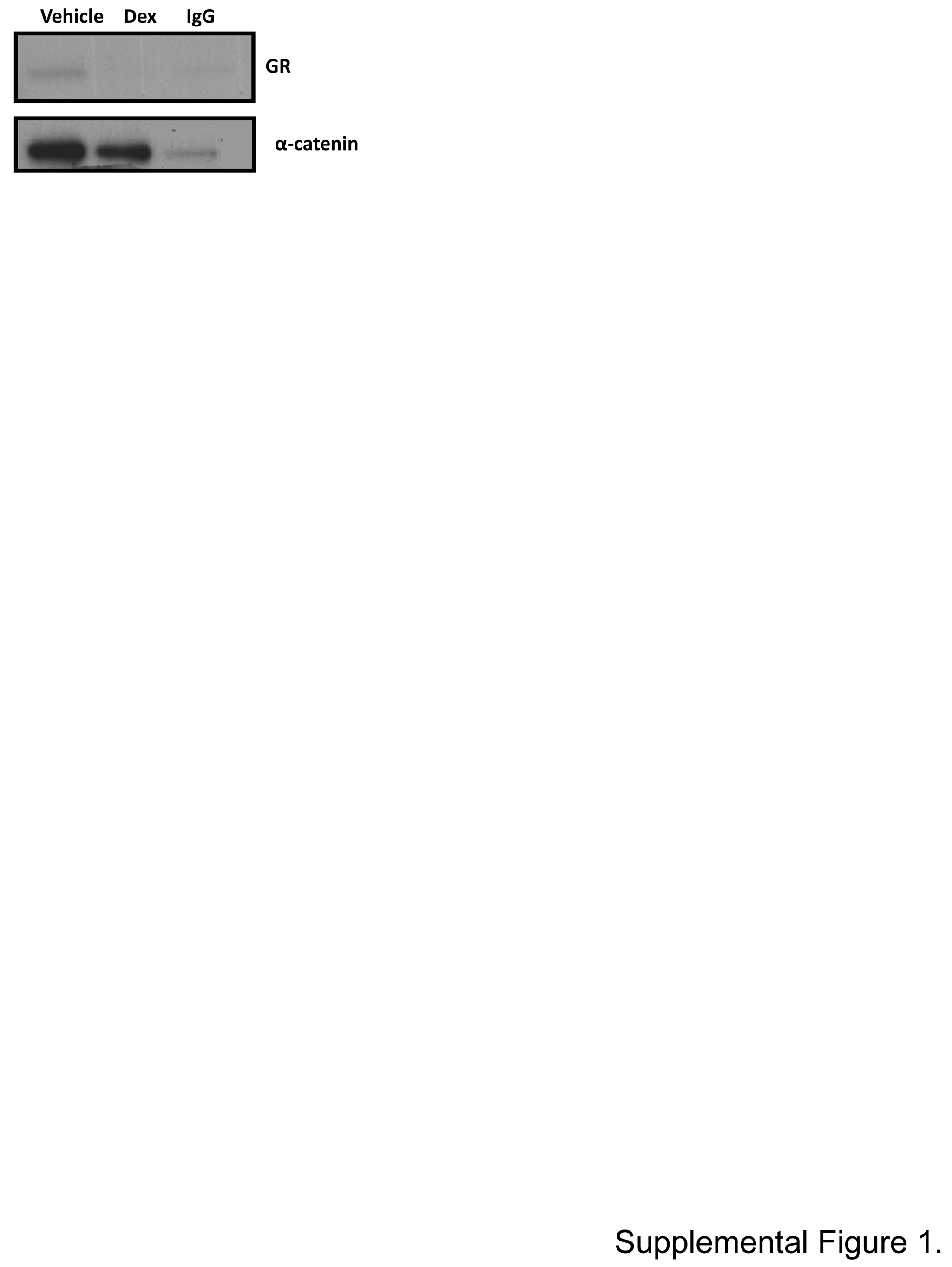

Supplement: Figure S1 — Immunoprecipitation controls. Flow through from immunoprecitiptations shown in Figure 2 were blotted for the presence of GR and α-catenin As expected, no remaining GR was found in samples originating from cells incubated with Dex, whereas very little was found in vehicle treated samples. Alpha-catenin was found in both fractions. (TIF) [file pone.0063453.s001.tif]
